# Percutaneous versus surgical strategy for tracheostomy: protocol for a systematic review and meta-analysis of perioperative and postoperative complications

**DOI:** 10.1186/s13643-015-0092-5

**Published:** 2015-08-08

**Authors:** Rosa Klotz, Ulla Klaiber, Kathrin Grummich, Pascal Probst, Markus K. Diener, Markus W. Büchler, Phillip Knebel

**Affiliations:** Department of General, Visceral and Transplantation Surgery, University of Heidelberg, Im Neuenheimer Feld 110, 69120 Heidelberg, Germany; Study Center of the German Surgical Society (SDGC), University of Heidelberg, Im Neuenheimer Feld 110, 69120 Heidelberg, Germany

**Keywords:** Tracheostomy, Percutaneous dilatational tracheostomy, Surgical tracheostomy, Systematic review with meta-analysis

## Abstract

**Background:**

Tracheostomy is one of the most frequently performed procedures in intensive care medicine. The two main approaches to form a tracheostoma are the open surgical tracheotomy (ST) and the interventional strategy of percutaneous dilatational tracheotomy (PDT). It is particularly important to the critically ill patients that both procedures are performed with high success rates and low complication frequencies. Therefore, the aim of this systematic review is to summarize and analyze existing and relevant evidence for peri- and postoperative parameters of safety.

**Methods/design:**

A systematic literature search will be conducted in The Cochrane Library, MEDLINE, LILACS, and Embase to identify all randomized controlled trials (RCTs) comparing peri- and postoperative complications between the two strategies and to define the strategy with the lower risk of potentially life-threatening events. A priori defined data will be extracted from included studies, and methodological quality will be assessed according to the recommendations of the Cochrane Collaboration.

**Discussion:**

The findings of this systematic review with proportional meta-analysis will help to identify the strategy with the lowest frequency of potentially life-threatening events. This may influence daily practice, and the data may be implemented in treatment guidelines or serve as the basis for planning further randomized controlled trials. Considering the critical health of these patients, they will particularly benefit from evidence-based treatment.

**Systematic review registration:**

PROSPERO CRD42015021967

## Background

Tracheostomy is one of the most frequently performed procedures in intensive care medicine [1, 2]. In 2013, 38,800 tracheostomies were performed in Germany [3]. It is considered to be a safe technique to achieve sufficient ventilation for patients who suffer from an obstruction of the upper airway or who need long-term ventilation [4].

Two different strategies are available for the formation of a tracheostoma: first, described as early as 1909, open surgical tracheotomy (ST), which is predominantly performed by surgeons, and second, initially described in 1985 [5], the interventional strategy of percutaneous dilatational tracheotomy (PDT), which is performed by surgeons, internists, and anesthetists [6–8]. For the PDT, six different techniques that are commonly performed can be distinguished: balloon dilation tracheostomy (BDT), guide wire dilating forceps (GWT), multiple dilator tracheostomy (MDT), rotational dilation tracheostomy (RDT), single-step dilation tracheostomy (SSDT), and translaryngeal tracheostomy (TLT) [9].

Both the surgical and the percutaneous dilatational strategies provide advantages and disadvantages. Some complications which can compromise the airway, such as technical difficulties [9], paratracheal insertion, tracheal laceration [10–12], pneumothorax [13], loss of airway, and hemorrhage [14], tend to be unusual for the surgical procedure but more likely in the percutaneous procedure. Accordingly, the surgical strategy appears to be beneficial with regard to the airway safety. In contray, stoma inflammation and infection might be more rare when PDT is performed [9, 15].

ST immediately secures the newly established access to the airway. In case of a dislodged tracheal cannula, a re-intubation is easily possible. In contrast, the PDT is performed via a small skin incision followed by different dilatational mechanisms and insertion of the tracheal cannula. Disadvantageously, in case of accidental dislocation of the tracheal cannula, a re-intubation via the newly achieved access might not be possible. Thus, the life-threatening situation that the patient cannot be safely ventilated might occur.

However, time, effort, inconvenience [9, 16], and the need to transport the critically ill patients to the operation theater (which is not necessary for the percutaneous strategy) have been arguments against ST. But by now, it has been demonstrated that both PDT and ST can be safely performed at the bedside by experienced, skilled practitioners [17].

Numerous common late complications are observed equally often with both strategies, such as tracheal stenosis, tracheal fistulas, and tracheomalacia [9, 18, 19].

Consequently, there is disagreement regarding which strategy offers the superior benefit/risk ratio for critically ill patients. Previously published meta-analyses reported conflicting results [2, 6, 7, 9, 20, 21], though some older reviews have not included the currently available percutaneous techniques. None of the existing reviews succeeded in including all existing, relevant randomized controlled trials (RCTs). Additionally, since the last published meta-analysis in 2014 [9], two recent RCTs with 140 additional patients have been published.

Until now, the choice of strategy is primarily made by the preference of the surgeon and not by evidence, as evidence for the strategy with the best parameters of safety is poor and conflicting.

## Methods/design

The protocol of this study is written according to the Preferred Reporting Items for Systematic Reviews and Meta-Analysis Protocols 2015 (PRISMA-P 2015) [22]. The following methods will be applied.

### Systematic literature search methodology

A systematic literature search will be conducted to identify all relevant RCTs comparing ST and PDT. Database searches will be done in the Cochrane Library (CENTRAL), PubMed/MEDLINE, LILACS, and Embase using the following search terms: Tracheotomy/ Tracheostomy /percutaneous/ dilatational/ conventional/open/ surgical. Search terms will be built into effective search strategies using Medical Subject Headings (MeSH) terms in combination with free text terms and Boolean operators customized for each database. The clinical trials register ClinicalTrials.gov will also be searched to identify potentially unpublished or ongoing trials in this field. Furthermore, experts in this field will be asked for further relevant trials.

Published and unpublished RCTs will be searched and no language restrictions will be applied. If relevant, non-English language papers will also be included and translated. All articles will be entered into a library in the reference software program EndNote (EndNote X7, Philadelphia).

### Study selection

Two review authors will independently screen all records of potential trials. If the title and abstract suggest relevance, records will be coded as “retrieve” (eligible or potentially eligible/unclear). The full-text study reports/publications of those abstracts will be assessed for eligibility by the same two review authors independently. Any disagreements will be resolved through discussion or, if required, consultation of a third person to reach consent about which studies will be included for review. Duplicates and collate multiple reports of the same trial will be identified and excluded so that each trial rather than each report is the unit of interest in the review. The selection process will be recorded in sufficient detail to complete a PRISMA flow diagram and characteristics of excluded trials table.

### Data extraction and management

For extraction of trial characteristics and outcome data, a standardized data collection form will be used. This form will be pre-tested on three trials in the review before application on the whole set of included trials. Two review authors will extract data from included trials. The following trial characteristics will be extracted:

1. Methods: author, year and journal of publication, trial design (registered: yes/no; number of treatment arms), total duration of the trial, number of trial centers and location (mono-center/multicenter, participating countries), sample size calculation (yes/no/not stated, power and delta in %), number of and reason for withdrawals, follow-up time (in days/not stated).
2. Participants: mean age, gender, body mass index, diagnosis (underlying disease), anatomical cervical variance (yes (what kind of)/no/not stated), prior cervical surgery (yes/no/not stated), inclusion criteria, exclusion criteria
3. Interventions: performers’ discipline (surgeon/non-surgeon/not stated) and experience (addressed: yes/no/in which way) technical details of intervention (description of technique, location (ICU/OR/not stated), presence of bronchoscopic or sonographic guidance (yes/no/not stated))
4. Outcomes: primary and secondary outcomes specified and collected, exact definitions of outcomes reported (yes/no)
  Potentially life-threatening events: loss of airway, false route, tracheal/esophageal injury, major bleeding, gastric aspiration, pneumothorax/mediastinum, subcutaneous emphysema, difficult tube change, other potentially life-threatening events
  Mortality
  Stomal inflammation (defined as edema and/or erythema and/or tenderness)
  Stomal infection (signs of inflammation and (possibly culture-positive) purulent discharge (possibly requiring antibiotic therapy))
  Late complications: tracheal stenosis, tracheomalacia, tracheocutaneous/esophageal fistula
  Duration of procedure
  Major complications (mortality, technical difficulties leading to life-threatening events, loss of airway, false route, tracheal/esophageal injury, major bleeding, gastric aspiration, pneumothorax/-mediastinum, subcutaneous emphysema, difficult tube change, tracheal stenosis, tracheomalacia, tracheocutaneous/-esophageal fistula)
  Minor complications (technical difficulties without life-threatening event, cuff leak, hypoxemia, stomal inflammation, stomal infection, minor bleeding)
5. Notes: funding for trial (industry/independent/not stated/name of sponsor), notable conflicts of interest of trial authors (stated/not stated/how described)

All disagreements of the two review authors will be resolved by consensus or by involving a third person.

### Endpoints of the study

The combined primary endpoint will be the risk of potentially life-threatening events including loss of airway, false route, tracheal/esophageal injury, major bleeding, gastric aspiration, pneumothorax/-mediastinum, subcutaneous emphysema, difficult tube change, other potentially life-threatening events. All potentially life-threatening events will be added to calculate the combined endpoint. For each procedure, not more than one potentially life-threatening event will be counted to estimate the combined primary endpoint.

Secondly, mortality intraoperatively and during the first 24 h after the intervention will be assessed. Furthermore, complications such as technical difficulties, stomal infection, stomal inflammation, and late complications will be evaluated separately as well as the duration of the procedure.

### Subgroup analysis

To analyze which PDT technique is favorable, to assess the concomitant diagnostic (bronchoscopy/ultrasound), to examine the profession and experience of the performing physician, and to evaluate the location of performance, the following subgroup analyses are planned to be performed, if sufficient data are available:

1. BDT versus ST
2. GWT versus ST
3. MDT versus ST
4. PDT versus ST
5. RDT versus ST
6. SSDT versus ST
7. TLT versus ST
8. Antegrade techniques (BDT, GWT, MDT, PDT, SSDT) versus ST
9. Bronchoscopic or sonographic guided tracheostomy versus no visual control during tracheostomy
10. Tracheotomy performed by surgeons versus tracheotomy performed by other physicians
11. PDTs performed on the intensive care unit versus in the operating theater
12. STs performed on the intensive care unit versus STs in the operating theater

### Assessment of the methodological quality of included studies

The risk of bias will be assessed for each study using the criteria outlined in the *Cochrane Handbook for Systematic Reviews of Interventions* [23]. Any disagreement will be solved by discussion or by involving a third reviewer. Each of the following domains will be evaluated:

1. Random sequence generation
2. Allocation concealment
3. Blinding of participants and personnel
4. Blinding of outcome assessment
5. Incomplete outcome data
6. Selective outcome reporting
7. Other bias (e.g., baseline imbalance, early termination of the trial, funding bias)

Each potential source of bias will be graded as high, low, or unclear and a quote from the study report together with a justification for the judgment will be presented in the risk of bias table. In case of information of unpublished data or correspondence with an author of an included trial, this will be noted in the risk of bias table. The quality of the body of evidence will be addressed using the specific evidence grading system developed by the GRADE collaboration. The following five considerations will be included: limitations in the design, indirectness of evidence, unexplained heterogeneity or inconsistency of results, imprecision of results, and high probability of publication bias [24].

To assess the robustness of our conclusions, sensitivity analysis will be conducted excluding studies with less than the average number of positive judgements in the risk of bias assessment.

The review will be conducted according to the published protocol, and any deviations from it will be reported in the “Differences between protocol and review” section of the systematic review. In case of missing data, investigators or study sponsors will be contacted.

### Statistical analysis

Dichotomous data will be analyzed as absolute differences with their 95 % confidence intervals and continuous data as mean difference. Meta-analyses will only be performed where this is meaningful, i.e., if the treatments, participants, and the underlying clinical question are similar enough to justify pooling.

In the case of continuous data, means and standard deviations will be reported. When medians and interquartile ranges are reported, the methods described by Higgins [23] and by Hozo [25] to calculate means and standard deviations will be used. The decision to conduct quantitative synthesis with these data will be based upon individual decision for each outcome.

Whenever sufficient data for a specific outcome are provided, meta-analysis will be performed by the use of a random effects model [26]. If substantial heterogeneity (>75 %) is identified, this will be explored by pre-specified subgroup analyses.

### Assessment of heterogeneity

*I*^2^ statistics will be used to measure statistical heterogeneity among the trials in each analysis. An *I*^2^ < 25 % will be considered a low heterogeneity and an *I*^2^ > 75 % will be considered a high heterogeneity. If there is an extreme level of heterogeneity, summary effect measures will be interpreted with caution. Clinical heterogeneity will be explored by assessing differences in baseline data, performers’ discipline, type of percutaneous technique, definitions of outcome parameters, and operative and/or perioperative management. The presence of strong clinical heterogeneity will be considered in the decision to conduct quantitative synthesis of data or to perform sensitivity analyses with a special focus.

Trial authors will be contacted asking them to provide missing outcome data if this is necessary. Where this will be not possible, and the missing data is thought to introduce serious bias, the impact of including such studies in the overall assessment of results will be explored by a sensitivity analysis. A funnel plot will be created and examined for asymmetry to explore possible publication bias.

## Discussion

Most patients undergoing tracheostomy are critically ill and even minor complications of the intervention can lead to severe consequences in their already unstable health condition. Thus, even small differences between the two compared strategies seem to be relevant for both patients and the healthcare system, even more when the commonness of this intervention is taken into account.

A combined primary endpoint was chosen for this study assessing the potentially life-threatening events related to the tracheostoma formation, as the overall risk of a possibly lethal situation of the intervention was found to be the most clinically and patient-relevant parameter. Addressing several single complications of the intervention could not satisfactorily answer the question, “Which strategy is more beneficial?” Our chosen, clinically relevant endpoint has not been used in any of the previous meta-analyses before, and furthermore, all currently available studies have not been evaluated.

To achieve the highest possible level of evidence, this systematic review and proportional meta-analysis will include RCTs only. Assessment of methodological quality will be performed according to the recommendations of the *Cochrane Handbook* and the five GRADE’s considerations will be applied.

Subanalysis of the different PDT techniques will help to determine if any of the percutaneous dilatational methods should not be considered anymore or if another technique is preferable over the others. Patients undergoing emergency tracheostomy and children will not be included, so that they do not introduce heterogeneity. Furthermore, because of the relevance and great acceptance of the two strategies investigated, we still expect a sufficient sample size in both study groups.

In case of low sample sizes in the different groups of the various PDT techniques described above, the techniques will be grouped in ante- and retrograde techniques. Thus, at least for these formed groups of PDT techniques, valid results are achievable.

To date, there is no gold standard for the strategy of tracheostomy as evidence is poor. The findings of this systematic review with proportional meta-analysis will help to compare potentially life-threatening event rates and procedure-related mortality in the two main approaches. This may influence daily practice and the data may be implemented in treatment guidelines. Considering the clinical impact of tracheotomy on critically ill patients, they will particularly benefit from evidence-based treatment.

## Abbreviations

BDT: balloon dilation tracheostomy
GRADE: Grades of Recommendation, Assessment, Development and Evaluation Working Group
GWT: guide wire dilating forceps tracheostomy
MDT: multiple dilator tracheostomy
MeSH: Medical Subject Headings
PDT: percutaneous dilatational tracheostomy
PRISMA-P 2015: Preferred Reporting Items for Systematic Reviews and Meta-Analyses for Protocols 2015
RCT: randomized controlled trial
RDT: rotational dilation tracheostomy
SSDT: single-step dilation tracheostomy
ST: surgical tracheotomy
TLT: translaryngeal tracheostomy
